# Contribution of the Local RAS to Hematopoietic Function: A Novel Therapeutic Target

**DOI:** 10.3389/fendo.2013.00157

**Published:** 2013-10-23

**Authors:** Kathleen E. Rodgers, Gere S. diZerega

**Affiliations:** ^1^School of Pharmacy, University of Southern California, Los Angeles, CA, USA; ^2^US Biotest, Inc., San Luis Obispo, CA, USA; ^3^Keck School of Medicine at USC, Los Angeles, CA, USA

**Keywords:** renin-angiotensin system, bone marrow, stem cells, angiotensin 1-7, CD143, hematopoiesis, myelosuppression

## Abstract

The renin-angiotensin system (RAS) has long been a known endocrine system that is involved in regulation of blood pressure and fluid balance. Over the last two decades, evidence has accrued that shows that there are local RAS that can affect cellular activity, tissue injury, and tissue regeneration. There are locally active ligand peptides, mediators, receptors, and signaling pathways of the RAS in the bone marrow (BM). This system is fundamentally involved and controls the essential steps of primitive and definitive blood-cell production. Hematopoiesis, erythropoiesis, myelopoiesis, thrombopoiesis, formation of monocytic and lymphocytic lineages, as well as stromal elements are regulated by the local BM RAS. The expression of a local BM RAS has been shown in very early, primitive embryonic hematopoiesis. Angiotensin-converting enzyme (ACE-1, CD143) is expressed on the surface of hemangioblasts and isolation of the CD143 positive cells allows for recovery of all hemangioblast activity, the first endothelial and hematopoietic cells, forming the marrow cavity in the embryo. CD143 expression also marks long-term blood-forming CD34+ BM cells. Expression of receptors of the RAS is modified in the BM with cellular maturation and by injury. Ligation of the receptors of the RAS has been shown to modify the status of the BM resulting in accelerated hematopoiesis after injury. The aim of the present review is to outline the known functions of the local BM RAS within the context of primitive and definitive hematopoiesis as well as modification of BM recovery by administration of exogenous ligands of the RAS. Targeting the actions of local RAS molecules could represent a valuable therapeutic option for the management of BM recovery after injury as well as neoplastic disorders.

## Introduction

The first evidence that there are effects of the renin-angiotensin system (RAS) on bone marrow (BM) and hematopoiesis resulted from clinical use of therapeutics that modify the production/action of angiotensin II (A-II). From the early 1980s, studies showed that there was a small reduction in hematocrit in patients receiving angiotensin-converting enzyme (ACE) inhibitors, particularly with enalapril (1). Further, enalapril use was associated with anemia in renal transplant patients as well as normal rats (2, 3). Prior to the use of Epogen to treat anemia in patients on long-term dialysis, the majority of patients receiving captopril developed dose dependent anemia that was reversed upon discontinuation of captopril treatment (4). Erythrocytosis occurs after renal transplantation. In these patients, there are anecdotal reports and studies showed that ACE inhibitors, and angiotensin receptor blockers (ARBs) reduce hematocrit levels in post-transplantation erythrocytosis (5–7). In two prospective studies, a reduction in hemoglobin concentrations was reported in hypertensive patients treated with ARBs compared with patients treated with β-blockers or calcium antagonists (8, 9). These observations led to the hypothesis in the mid-1990s that there is a local RAS in the BM that is involved in the regulation of hematopoiesis. These early observations were thought to be the result of increased levels of an inhibitor of hematopoiesis, the acetylated tetrapeptide AcSKPD, that is hydrolyzed by ACE or due to interactions of the RAS directly with the hematopoietic system. Activation of the RAS was shown to enhance erythropoietin production (10). Similarly, administration of ACE inhibitors reduces plasma erythropoietin levels, exacerbating anemia (11). Administration of A-II to patients after hemorrhage leads to an increase in plasma erythropoietin levels (12). This work was recently reviewed by Vlahakos (13). All of these studies suggested that the RAS directly modifies erythropoiesis.

## The Renin-Angiotensin System in Bone Marrow and Hematopoiesis

### RAS components and RAS knockout mice

**Figure 1:**
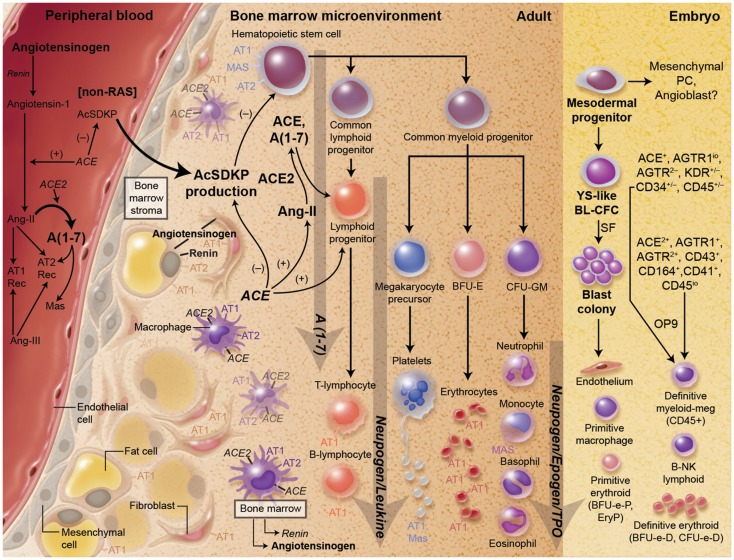
**There is substantial evidence for a key role for the RAS both primitive and definitive hematopoiesis as well as the development of hematopoietic progenitor cells**. Every component of the RAS is present in the local environment of the bone marrow.

Research over the last few decades has confirmed the presence of a local, integrated RAS within several tissues. Every known component of the RAS is contained within BM cells, including mRNA for angiotensinogen, renin, ACE, AT_1a_, AT_2_, Mas, and ACE_2_ [(14, 15), Figure 1]. Transgenic mice carrying both human renin and angiotensinogen genes have increased hematocrits in animals intact for AT1, but not AT1 null animals (16). Further, mice that are deficient in individual components of the RAS have been shown to have impairment of hematopoiesis. Direct evidence for the role of RAS in hematopoiesis independent of ACE hydrolysis of AcSDKP came from animal models (ACE Knock out (KO)) and *in vitro* studies. In one strain of ACE-KO mice, there were increases in circulating levels of AcSDKP that was accompanied by a 35 mm Hg decrease in blood pressure, renal insufficiency, and unexpected normocytic anemia associated with an increase in circulating erythropoietin in response to anemia (17). A similar anemia was also present in another strain of genetically engineered mice expressing a truncated form of secreted ACE (18). In these mice, plasma ACE activity was reduced without evidence of renal insufficiency indicating that the anemia was not the consequence of the renal failure, but the result of a reduction in red-cell mass. The degree of anemia in these two mouse strains was similar, despite a significant difference between plasma AcSDKP levels. This finding again suggests that AcSDKP was not the primary cause of anemia. To evaluate the role of A-II in hematopoiesis, hematocrit was measured before and after A-II infusion for 2 weeks. The hematocrit level was corrected in ACE-deficient mice to near wild-type levels, strongly suggesting that the lack of A-II in these mice was the direct cause of the anemia. Further, several studies have shown that A-II, in the presence of erythropoietin, will increase erythroid progenitors *in vitro* (19–21).

In another strain of ACE-KO mice, abnormalities in myelopoiesis were seen. These abnormalities were characterized by increased BM myeloblasts and myelocytes, as well as extramedullary myelopoiesis (22). Further, neutrophils (banded and segmented) and erythroid elements were reduced approximately one third in the BM. Increases in splenic CD11b+ Gr1dim cells in these mice together with the increase in BM myelocytes indicate a block in myeloid differentiation at the post-GMP stage of development. A-II, through AT1, stimulates myeloid differentiation and function. In these mice, plasma levels of A-II were decreased by approximately 10-fold. Part of these effects was thought to be due to up-regulation of C/EBPα, a central transcription factor of myelopoiesis. Macrophages derived from ACE-KO mice had depressed C/EBPα expression and treatment with A-II restored expression of this transcription factor (23). Function of the myeloid cells that did develop in these mice was also impaired (22). Peritoneal macrophages from ACE-KO mice were deficient in the production of effector molecules, such as tumor necrosis factor-α, interleukin-12p40, and CD86 when stimulated with lipopolysaccharide and interferon-γ. ACE-KO mice were more susceptible to *Staphylococcus aureus* infection showing a reduction in host resistance.

### RAS in hematopoiesis

Observations in KO mice provides evidence that points to a significant role for the RAS in the regulation of hematopoiesis and the development of hematopoietic progenitor cells (23–25). The central role of the RAS in regulating early hematopoiesis has been the focus of several reviews (23, 26–28). In addition to reduced/delayed hematopoiesis in ACE-KO mice, is the observation that expression of ACE demarcates early events in hematopoiesis both in the fetus and the adult. The earliest marker for the isolation of hemangioblasts, hematopoietic stem cells, and epidermal stem cells is ACE_1_ (CD143) (26, 29–31). ACE_1_ was also shown to be expressed in all presumptive and developing blood-forming tissues of the human embryo and fetus: para-aortic splanchnopleura, yolk sac, aorta-gonad-mesonephros, liver, and BM (32). This is expanded upon further below.

The role of ACE and A-II in adult hematopoiesis led to investigations of the possible role of the RAS in primitive hematopoiesis. The first studies were done in avian models. In these models, primitive hematopoiesis occurs prior to intra-embryonic hematopoiesis (33). Extra-embryonic blood islands differentiate into endothelial and hematopoietic cells (34). ACE gene expression and protein was detected in the yolk-sac endoderm before the beginning of blood-island differentiation, when the circulation is not yet established between the yolk sac and the embryo (35). At 30 h development in the avian model, the other components of the RAS, renin and angiotensinogen, were present in the vicinity of the blood islands, which strongly suggests a role for the RAS in erythropoiesis. Administration of ACE inhibitors or ARBs to 2-day-old embryos resulted in a significantly lower hematocrit in treated embryos than in control embryos. These results show that the RAS modulates blood-island differentiation during the primitive yolk-sac erythropoiesis (35). Since these observations, studies were done to evaluate the expression of the RAS components in mammalian embryos, especially in humans. These studies confirm a role for RAS in mammalian primitive hematopoiesis.

Studies in mammalian hematopoiesis were facilitated by the development of the monoclonal antibody, BB9. BB9 is specific for the somatic isoform of surface ACE (CD143). As outlined above, the first observation was that a protein immunologically similar to ACE is expressed in the embryonic para-aortic splanchnopleura, where blood-cell progenitors are generated (36), suggesting that a local RAS exists within the intra-embryonic sites of definitive hematopoiesis in the mammal. ACE expression was also identified emerging hematopoietic cells from both CD34^−^ and CD34^+^ areas of human yolk sac, intra-embryonic splanchnopleura, and hemogenic endothelium of the aorta-gonad-mesonephros region and fetal liver (FL) (29). The embryonic pattern of human ACE expression is consistent with a dorsal emigration of ACE^+^CD34^−^ hemangioblasts from the para-aortic splanchnopleura, and subsequent colonization of the ventral aspect of the dorsal aorta to give rise to CD34^+^ hemogenic endothelial cells. Using BB9, a primitive subset of CD34^+^ multi-lineage hematopoietic stem cell that could engraft NOD SCID mice was identified in adult BM, mobilized peripheral blood, and umbilical cord blood (37).

In 2008, Zambidis and others reported that ACE is a novel marker for identifying hemangioblasts differentiating from human embryonic stem cells (hESC). Cells developed from hESC that expressed ACE (ACE^+^CD45^−^CD34^±^) were hemangioblasts that were progenitors for not only endothelium but also both primitive and definitive human lymphohematopoietic cells. Thrombopoietin and basic fibroblast growth factor were identified as critical factors for the proliferation of human hemangioblasts. Furthermore, ACE and receptors for A-II, AT1, and AT2 directly regulated hemangioblast expansion and differentiation. ACE enzymatic activity was required for hemangioblast expansion. Further, differentiation toward either endothelium or multipotent hematopoietic progenitors is modified by exposure to AT1 and AT2 antagonists. In this study, AT2 function was necessary for expansion of hemangioblast colonies into multipotent hematopoietic progenitors; whereas blockade of AT2 by PD 123319 abolished hematopoietic differentiation and sent the hemangioblasts toward an endothelial lineage. Conversely, inhibition of AT1 by losartan augmented differentiation of hemangioblast colonies into multipotent hematopoietic progenitors.

The influence of modification of the RAS in mesenchymal stem cells (MSC) has also been evaluated (38). Expression of the AT1 receptor in MSC was initially observed in 2000 (21). MSC can be differentiated into a number of cell types, including those involved in fibrosis and adipogenesis. Differentiation of human MSCs into adipocytes resulted in increased expression of renin and AT2 and a decrease in angiotensinogen and ACE expression with a net increase in endogenous cellular A-II production. Incubation of MSC with A-II with and without an AT1 antagonist inhibited adipogenesis, whereas A-II and an AT2 antagonist abolished the inhibition of adipogenesis. MSC can also be differentiated into renin-producing cells in the kidney, juxtaglomerular cells (39, 40). Increased numbers or activation of these cells is important in the initiation of pathological effects of chronic overexpression of A-II through increase production of renin, the rate-limiting enzyme in the production of A-II.

## RAS: A Novel Target in Bone Marrow Injury and Myelosuppression

Multi-lineage suppression of BM progenitors occurs following myelosuppressive chemotherapy, as well as radiation, resulting in cytopenias of their formed elements in the peripheral circulation. Myelosuppression and the more severe myeloablation (requiring hematopoietic stem cell support for recovery) can be the result of exposure to chemotherapy or radiation therapy for neoplastic disease, to diagnostic radiation exposure or due to radiation exposure as a result of nuclear accident or terrorism. The manifestations of myelosuppression include anemia, thrombocytopenia, lymphopenia, and neutropenia. In the setting of chemo or radiation therapy, myelosuppression is often managed with a delay and/or a dose reduction in the next scheduled cycle of chemotherapy, to allow recovery of BM and circulating formed elements. However, such modifications to the chemotherapy regimen result in lower relative dose intensity (the ratio of delivered dose intensity to planned dose intensity). Numerous studies, particularly in breast cancer and NHL, have established that long-term survival may be compromised if the total dose or relative dose intensity falls below a threshold value (41–45).

Prolonged or severe myelotoxic effects may reflect a diminished hematopoietic reserve, which may occur with aging, age-related comorbidity, intensive chemotherapy, combined radiation therapy/radiation therapy, or previous exposures to myelosuppressive therapies. Therefore the risk of myelosuppression leading to modifications of chemotherapy is higher in older patients and patients with recurrent neoplasms. Even when the chemotherapy regimen is relatively benign and myelotoxicity is limited, elderly patients tend to be more vulnerable than younger patients (46).

The most widely utilized hematopoietic stimulants (erythropoietin, filgrastim, or sargramostim) act on later stage precursors and usually induce proliferation, differentiation, and mobilization of single cell lineages from the BM into the peripheral circulation. For this reason, they do not individually impact chronic and progressive multi-lineage cytopenias that commonly occur after myelosuppression. This finding suggests that a treatment which stimulates proliferation and differentiation of hematopoietic stem/progenitor cells should reduce incidence of clinically significant cytopenias.

## Angiotensin 1-7 as a Therapeutic in Bone Marrow Recovery

Because of the increased sensitivity of immature cells compared with more mature cells of a given cellular lineage to the proliferative effects of angiotensin peptides, therapeutic opportunities exist to enhance tissue regeneration, particularly the repair of injuries in the BM associated with chemotherapy and radiation. Potential populations include cancer patients receiving antineoplastic or radiation therapy with myelosuppressive side effects, stem cell transplant patients after myeloablative conditioning, patients with conditions resulting in ineffective myelopoiesis and apoptosis of hematopoietic progenitors and individuals exposed to nuclear radiation.

Peptides of the RAS are potent stimulators of progenitor cell proliferation (21, 24, 47–49). RAS receptors are increased after injury (50–53). Preclinical studies have shown that hematopoietic progenitor cells are sensitive to Angiotensin 1-7 [A(1-7)] stimulation and the effect of this biologically active member of the endogenous protective arm of the RAS *in vivo* is most robust after injury (24, 47–49, 54). A(1-7) has multi-lineage effects on hematopoietic progenitors *in vitro* and *in vivo* (24, 48, 52, 54–56) and is undergoing clinical development for the treatment of myelosuppression and to increase hematopoietic stem cell transplantation.

Angiotensin 1-7 treatment following 5-fluorouracil (5FU) administration resulted in a higher number of progenitors in the myeloid, megakaryocyte, and erythroid lineage in murine BM (48). However, the most extensive preclinical data set with A(1-7) is following myelosuppression due to total body irradiation (TBI) (24, 47). An early increase and sustained expansion in early mixed, myeloid, erythroid, and megakaryocytic progenitors was observed in A(1-7)-treated animals compared to controls. At 30 days after TBI, A(1-7) treatment increased early mixed progenitors (three- to fivefold), megakaryocyte (two- to threefold), myeloid (three- to sixfold), and erythroid (two- to fivefold) progenitors in the BM and reduced radiation-induced thrombocytopenia (RIT) (up to twofold). Consistent with clinical results below, it is important to initiate treatment with this peptide once the damage resulting from the myelotoxic exposure has ceased. For example, improvement in BM progenitors following TBI were better at higher doses of A(1-7) when drug was initiated at 48 h post-TBI as opposed to 24 h post-TBI.

In the A(1-7)-treated animals, the nadir in BM progenitors was not as low as in the control animals and accelerated recovery was observed. The multi-lineage BM response resulted in platelet and white blood-cell recovery after TBI (24, 47). Initiation of A(1-7) treatment could be delayed up to 5 days following TBI with continued improvement of hematopoietic recovery both in the BM and formed elements in the peripheral blood (24).

It is hypothesized that A(1-7) acts to stimulate hematopoiesis through the Mas receptor. The expression of Mas in normal BM is low. However, as with injuries to other tissues, Mas expression in hematopoietic progenitors in the BM is increased by myelosuppression (52). The ability of A(1-7) to accelerate BM recovery was blocked by administration of the Mas antagonist, A779; whereas losartan, an antagonist of AT1 (the constitutively expressed receptor for A-II) did not (24, 55). In order to ascertain the receptor that A(1-7) acts through to stimulate hematopoietic recovery, antagonists of the type I receptor (losartan), type 2 receptor (PD123319), or Mas receptor were co-administered with A(1-7). Administration of the antagonists had no effect on the recovery of platelets while the Mas antagonist blocked the acceleration of platelet recovery by A(1-7) (55). Of interest, RAS receptors, AT1, AT2, and Mas, are G coupled protein receptors that are capable of distinguishing small changes in peptide sequence and provide novel targets for modulation of hematopoiesis.

The kinetics of changes in hematopoietic progenitors in BM with A(1-7) treatment after TBI were evaluated. There was an early increase (up to fivefold by Day 7) in myeloid and erythroid progenitors that continued to expand more rapidly than in control animals through Day 14. The number of megakaryocytes in the BM was measured by CD41+ (platelet glycoprotein IIb of IIb/IIIa complex) expression. In contrast to myeloid and erythroid progenitors, the nadir for megakaryocyte number after TBI was Day 8. In control animals, recovery did not start until Day 14 and plateaued at Day 22. In the A(1-7)-treated animals, the nadir was not as low as the control animals and recovery was seen at Day 10. By Day 14, the megakaryocyte number doubled in the treated animals and was comparable to that observed in the control animals at Day 22. After Day 22 recovery in the control animals reached a plateau, and recovery of the number of megakaryocytes continued through Day 30 (the last time point measured) in the animals treated with A(1-7). Changes were also observed in thrombopoietin, a key regulator in platelet generation, production, and utilization that are consistent with these observations. At the nadir of platelet levels, there was increased utilization of thrombopoietin. However, later, when megakaryocyte levels and maturity were comparable to non-irradiated controls in A(1-7)-treated animals, there was an increase in circulating thrombopoietin levels. These data suggest that the primary action of A(1-7) is at the level of the progenitor cell.

In all studies with repeat bleeding, a nadir was found in platelet number after TBI even with A(1-7) treatment. However, the nadir was diminished at an early time point. It is hypothesized that the mechanism by which this occurs is A(1-7) reducing consumption of platelets as well as increasing their production. Platelet consumption occurs through bleeding at sites of injury due to radiation (such the gastrointestinal tract or the cerebral cortex) or through the formation of thrombus (in part due to endothelial dysfunction). As will be shown below, A(1-7) reduced mucosal injury. Further, A(1-7) reduces oxidative stress after injury, which would contribute to endothelial dysfunction.

## Synergy and Multi-Lineage Effects after Combining A(1-7) with Colony Stimulating Factors

Combining A(1-7) with commonly used growth factors [filgrastim (Neupogen^®^), and erythropoietin (Epogen^®^)], in C57Bl/6 mice post-chemotherapy increased the numbers of BM progenitor cells and formed elements in the peripheral circulation (52, 57). A(1-7) administered in combination with suboptimal doses of Neupogen^®^ throughout the post-myelosuppressive interval increased the number of progenitors and circulating WBC concentration to a greater extent than occurred with either drug alone (52). These studies indicate that the A(1-7) effects on progenitors can enhance the concentration of formed elements in the peripheral blood in the presence of appropriate differentiating agents.

Administration of A(1-7) with filgrastim (the latter given only 3 days starting at the white blood-cell nadir) decreased 10-fold the amount of filgrastim needed for optimal recovery of BM progenitors (CFU-GEMM, CFU-GM, CFU-Meg, and BFU-E) and circulating formed elements (WBC, platelets). In addition to the synergistic effects of combining A(1-7) and Neupogen on white blood-cell and neutrophil recovery, combining these therapies increased platelet concentrations above those observed with A(1-7) alone.

In addition, combination of A(1-7) with erythropoietin slightly increased (not significantly) red blood-cell concentrations above those achieved by erythropoietin alone. However, in this model, A(1-7) or A(1-7) in combination with erythropoietin increased all erythroid progenitors with the largest effect on early erythroid progenitors (immature BFU-E). As before with Neupogen, combining A(1-7) with Epogen has hematological effects outside of the erythroid lineage in that the concentration of circulating neutrophils was increased with this combination. In conclusion, filgrastim and erythropoietin acted synergistically with A(1-7) to increase the concentration of myeloid, megakaryocytic, and erythroid progenitor cells in the BM following chemotherapy, suggesting that A(1-7)’s multi-lineage effect on early progenitors in the marrow facilitates proliferation in response to lineage specific growth factors.

## Clinical Development of A(1-7) for Hematopoiesis

Multi-lineage suppression of marrow precursors occurs following myelosuppressive radiotherapy and chemotherapy resulting in cytopenias of one or more of the mature formed elements of blood. As described above, preclinical studies of A(1-7) demonstrated an increase in multiple lineages of early hematopoietic precursors from the BM and the peripheral blood of mice, and *in vitro* exposure of cells from human cord blood.

A Phase I/IIa prospective, blinded, randomized, dose-escalation study of a clinical formulation of A(1-7) was conducted in breast cancer subjects receiving three cycles of adjuvant doxorubicin and cyclophosphamide following surgical tumor reduction (54). The study compared the effects of up to 100 μg/kg of A(1-7) to filgrastim (*n* = 5) as a comparator arm for safety and response variables. A(1-7) was found to be safe and was well-tolerated. No dose-limiting toxicity was observed following subcutaneous administration of up to 100 μg/kg of A(1-7) for periods of up to 14 days. No A(1-7)-treated patients experienced any NCI Grade 1–4 platelet reductions compared to 60% of the controls. Further, patients had a lower incidence of lymphopenia, anemia, and mucositis. Additionally, following the completion of the third cycle of chemotherapy, recovery of hemoglobin, lymphocytes, leukocytes, neutrophils, and platelets was superior in A(1-7)-treated subjects. A(1-7) also reduced filgrastim use, as well as development of mucositis. Further, anemia was reduced even though two of the five control patients and one of 15 treated patients [in the lowest A(1-7) dose group] received erythropoietin for anemia. There were no A(1-7) treatment related serious adverse events (SAE) reported in this study.

A Phase 2b study evaluating the safety and efficacy of a clinical formulation of A(1-7) in reducing the incidence and severity of thrombocytopenia in subjects receiving a combination of gemcitabine and platinum therapy for ovarian carcinoma for six cycles was conducted. The primary endpoint of this study was the severity and incidence of thrombocytopenia as determined by the number of chemotherapy cycles during which the platelet count was below 50,000/mm^3^. A significant reduction of Grade 4 thrombocytopenia was seen in the 100 μg/kg group (56). In addition, there was a significant increase in the maximal percent increase in platelet count and delivery of scheduled chemotherapy dose on time in subjects treated with 100 μg/kg/day versus placebo-treated control. Maintenance of the scheduled chemotherapy at the desired dose has been linked with improved tumor control and long-term survival. No dose-limiting toxicity was observed during the course of the study and no investigational product-related SAEs or deaths occurred.

*Benefit:* data from clinical studies confirm and extend the results seen in preclinical models. These findings are consistent with the species homology of A(1-7). Overall, the observed increase in sensitivity of immature stem/early progenitor cells to the proliferative and regenerative effects of angiotensin peptides offers unique therapeutic opportunities including significantly enhanced hematopoietic recovery after chemotherapy as well as the potential to facilitate BM regeneration.

## Conclusion

A central role for the RAS in BM development and recovery has been the focus of two decades of research. ACE_1_ is a pivotal component in hematopoiesis in that expression marks early cells involved in primitive and definitive hematopoiesis. Reduction of ACE activity either through ACE inhibitors or ACE-KO results in abnormal hematopoiesis, particularly in the erythroid and myeloid lineages. While all components of the RAS are expressed in BM cells, fetal receptors of RAS, such as AT2, are expressed only on very early progenitors and are down regulated during differentiation. However, injury to the BM or myelosuppression modified RAS expression and up-regulates receptors of the protective RAS, Mas, and AT2.

Preclinical studies of A(1-7) suggest a potential to increase multiple lineages of early hematopoietic precursors cultured from the BM and the peripheral blood of mice. These studies have shown an increase in the number of progenitors and formed elements in the peripheral blood after treatment with A(1-7). Studies in murine models show that A(1-7) prevents or mitigates thrombocytopenia following myelotoxic chemotherapy. This benefit is supported by clinical data from multiple trials which shows decreased incidence of thrombocytopenia and increased BM recovery in A(1-7) treated patients following myelotoxic chemotherapy. Additional preclinical data in TBI models show that A(1-7) treatment also prevents or mitigates thrombocytopenia even when treatment initiation is delayed up to 48 h post exposure. It is anticipated that demonstration of the benefit of A(1-7) administration in these models will be translatable to humans for the indication of mitigating thrombocytopenia following TBI.

Clinical studies also suggest that exposure to A(1-7) maintains the pre-chemotherapy health of the BM by restoring the various hematopoietic parameters to baseline values and allows the maintenance of chemotherapy dose intensity (54, 56). This consistent A(1-7)-mediated return to baseline hematopoietic values may be due to an increase in the number of hematopoietic progenitor cells thereby pharmacologically replenishing the hematopoietic system.

## Conflict of Interest Statement

Dr. Rodgers and Dr. diZerega have conflicts. They are inventors of patents covering the activities of A(1-7) in bone marrow recovery.
